# Optical map guided genome assembly

**DOI:** 10.1186/s12859-020-03623-1

**Published:** 2020-07-06

**Authors:** Miika Leinonen, Leena Salmela

**Affiliations:** Department of Computer Science, Helsinki Institute for Information Technology, University of Helsinki, Pietari Kalmin katu 5, Helsinki, Finland

**Keywords:** Genome assembly, Optical mapping

## Abstract

**Background:**

The long reads produced by third generation sequencing technologies have significantly boosted the results of genome assembly but still, genome-wide assemblies solely based on read data cannot be produced. Thus, for example, optical mapping data has been used to further improve genome assemblies but it has mostly been applied in a post-processing stage after contig assembly.

**Results:**

We propose OpticalKermit which directly integrates genome wide optical maps into contig assembly. We show how genome wide optical maps can be used to localize reads on the genome and then we adapt the Kermit method, which originally incorporated genetic linkage maps to the miniasm assembler, to use this information in contig assembly. Our experimental results show that incorporating genome wide optical maps to the contig assembly of miniasm increases NGA50 while the number of misassemblies decreases or stays the same. Furthermore, when compared to the Canu assembler, OpticalKermit produces an assembly with almost three times higher NGA50 with a lower number of misassemblies on real *A. thaliana* reads.

**Conclusions:**

OpticalKermit successfully incorporates optical mapping data directly to contig assembly of eukaryotic genomes. Our results show that this is a promising approach to improve the contiguity of genome assemblies.

## Background

The long reads produced by third generation sequencing technologies such as Pacific Biosciences and Oxford Nanopore have enabled large improvements in *de novo* genome assembly. Nevertheless, assemblies produced solely on read data are not complete and typically contain orders of magnitudes more contigs than the sequenced organism has chromosomes. To further improve these assemblies, several long-range technologies such as optical mapping, genetic linkage maps, and Hi-C based analysis have been developed [1].

Here we concentrate on using optical mapping data to improve genome assembly. Optical maps are produced by fragmenting the genome to produce hundreds of kilobases long DNA molecules. Each DNA molecule is then elongated on a plate. A restriction enzyme which cuts at a specific DNA motif is applied on the DNA molecules and the order and length of the resulting fragments are measured by imaging [2, 3]. This results in raw optical mapping data which is then assembled to genome-wide optical maps.

Nowadays the optical mapping data is commonly utilized after contig assembly to further scaffold the contigs. We are aware of only two works attempting to use optical mapping data during contig assembly: AGORA [4] and KOOTA [5]. These tools were tested only on small genomes but the experiments showed that integrating optical mapping data to contig assembly can be beneficial.

Here we present OPTICALKERMIT, a contig assembler using both Pacific Biosciences sequencing reads and a genome-wide optical map. Similar to KOOTA [5], we first locate the reads on the genome-wide optical map. Whereas KOOTA mapped the in silico digested reads directly to the optical map, we first create preliminary contigs and use them to get more accurate location information for the reads. Finally, we assemble the reads augmented with approximate location information using the guided assembly approach developed in Kermit [6].

Our experiments show that using the genome-wide optical map increases the NGA50 of the assembled contigs as compared to assembling just the reads with the same assembly method. Furthermore, the number of misassemblies decreased or stayed the same. When compared to the Canu assembler [7] on real *A. thaliana* reads, OPTICALKERMIT produces an assembly with almost three times higher NGA50 and a lower number of misassemblies.

OPTICALKERMIT is freely available at https://github.com/Denopia/kermit-optical-maps.

### Related work

**Fig. 1:**
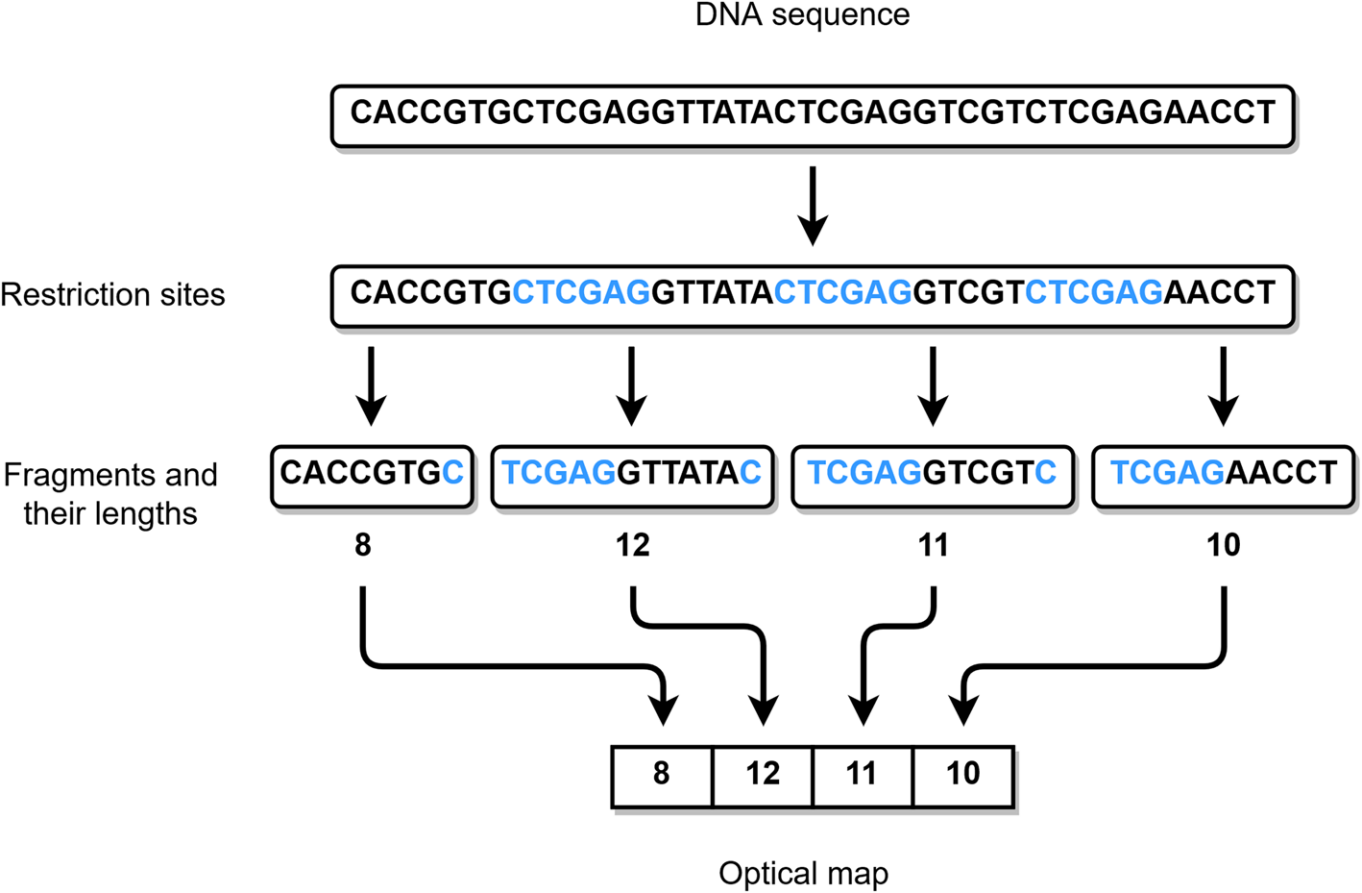
Example DNA sequence and its optical map corresponding to XhoI restriction enzyme. XhoI recognizes ‘CTCGAG’ sites and cleaves the sequence after the first C-nucleotides. The lengths of the resulting fragments are then measured to compose the optical map

An optical map is a sequence of lengths of subsequent DNA sequence fragments. A DNA sequence can be cut at specific places, *restriction sites*, using *restriction enzymes*. Applying a restriction enzyme to a DNA molecule cuts it into fragments at the corresponding restriction sites. For example, the enzyme XhoI recognizes the nucleotide sequence ‘CTCGAG’, and cuts the DNA molecule after the first C-nucleotide. This process leaves us with a number of consecutive DNA fragments, whose order is known, and their length can be measured. The measured lengths put together in order give us an optical map of the DNA sequence. A simplified example of a DNA sequence and its optical map can be seen in Fig. 1. The optical map of a genome we want to assemble is usually generated this way in a laboratory environment. In the case we have access to the DNA sequence itself, we can use in silico digestion. This means that the sequence is fragmented computationally by finding each occurrence of a subsequence corresponding to a restriction enzyme and cutting it at these sites. For example, optical maps for reads and contigs can be acquired this way since their sequences are known.

A basic task in processing optical mapping data is to align the optical maps against each other or to align in silico digested contigs to an optical map. Work in this area was pioneered by Valouev et al. [8] who developed a dynamic programming algorithm to solve the alignment problem. A similar approach was later used in SOMA [9]. Because of the quadratic time complexity, these approaches can be slow. Thus several methods have been developed to align optical mapping data more efficiently. OMBlast [10] uses a seed and extend approach for alignment. Maligner [11] offers two alignment modes: a sensitive mode based on dynamic programming and an efficient indexing based approach that tolerates unmatched cutting sites in the reference but not in the query optical map. TWIN [12] uses an FM-index to facilitate efficient alignments, whereas KOHDISTA [13] indexes the optical maps as an automaton. Once in silico digested contigs have been aligned to a genome-wide optical map, the alignments can be used to detect misassemblies [14] or to order the contigs into scaffolds [15].

Optical maps have been used in several genome projects to improve the contiguity of the assembly, see e.g. [16–21]. However, optical mapping data has usually been used in a post-processing step after the contigs have been constructed. Some preliminary research has been done to involve optical maps already during the contig assembly process. For example, AGORA [4] is an assembler program that can utilize optical maps, but it was only tested with error-free reads of very small bacterial genomes. Nevertheless, AGORA got positive results for using optical maps. Another example program called KOOTA [5] also takes advantage of optical maps during assembly. While KOOTA did not perform competitively compared to the other current assemblers, it demonstrated that optical maps can be used to improve specific phases of the assembly process.

In our approach, we will use Kermit [6] to implement a guided assembly. Kermit was initially developed for genetic linkage maps. A genetic linkage map consists of a set of markers, e.g. SNVs, on a genome. Typically the markers are divided into chromosomes and within each chromosome, the markers are further divided into ordered bins. The markers of a genetic linkage map are derived from a sequenced cross which is a population of related individuals. The markers are then assigned to chromosomes and bins within chromosomes based on the observed hereditary patterns. The bins are ordered, which means that if two markers are in different bins, we know which marker appears before the other in the genome, while nothing can be said about the relative order of markers within a single bin. Kermit assigns colors to bins, represented as integers starting from 0. If the integer, in other words, a color, of a bin is smaller than another bin’s, we know that all markers within it appear before the markers in the other one. Markers in a bin are given the color of the bin they reside in. Kermit takes as input a set of sequencing reads and a genetic linkage map. It then maps the markers of the genetic linkage map to the reads and assigns reads to bins based on these mappings. During contig assembly, the assignment of reads to bins is used to produce longer contigs than would be possible solely based on the reads.

## Results

### Overview of our method

Our method takes as input high error rate third generation sequencing reads such as those produced by the Pacific Biosciences sequencing technology and a genome-wide optical map of the target genome. Our aim is to align the reads to the optical map and then use this location information to perform guided genome assembly. The approximate location information of reads will be expressed as colors. In our case, each fragment of the genome-wide optical map has its own color. Thus after the reads have been aligned to the genome-wide optical map, we know which fragments they overlap and we can color them with the corresponding colors. The Kermit assembler [6] can then use this color information for guided genome assembly and thus produce contigs that represent the reference genome more accurately.

To align the reads to a genome-wide optical map, two problems need to be overcome: (i) many of the cutting sites have been confounded in the reads because of the high abundance of sequencing errors and (ii) the reads are too short to unambiguously align directly to the genome-wide optical map.

**Fig. 2:**
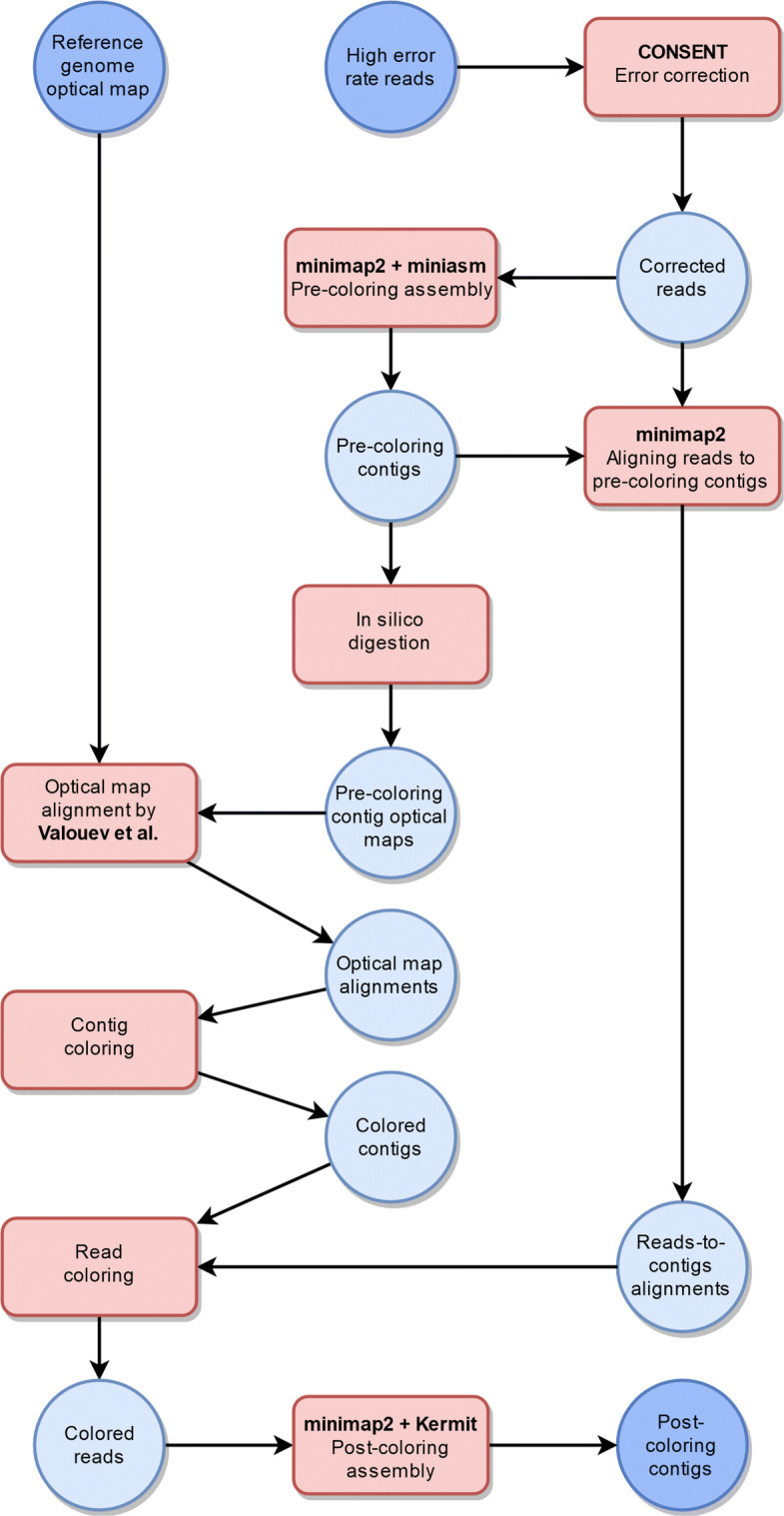
OPTICALKERMIT assembly workflow

To solve the first problem, we use CONSENT [22] to correct the reads. To solve the second problem, we use the corrected reads directly with a *de novo* assembler to acquire *pre-coloring contigs*, contigs that are assembled using non-colored reads. After this in silico digested optical maps of the pre-coloring contigs are created and aligned to the reference genome optical map. With optical map alignments, we are able to approximate the locations of the pre-coloring contigs within the reference genome and color them accordingly. Most of the contigs can be colored because they are much longer and their optical maps have more fragments compared to the reads. Next, the reads are aligned to the pre-coloring contigs. Alignments contain information on how the reads and contigs overlap, i.e. which contig a read aligns with and in which positions the overlap starts and ends. With this information and colored pre-coloring contigs, the reads can be colored. Afterward, the colored reads are ready to be given to the Kermit assembler as input. Kermit outputs new *post-coloring contigs*, which are the final product of our guided genome assembly, representing the reference genome more accurately than the pre-coloring contigs. The whole assembly workflow is shown in Fig. 2.

### Datasets

We used three different sets of reads to test our guided genome assembly pipeline. One set contained reads of *A. thaliana* obtained by PacBio sequencing. The two others were simulated reads of *S. cerevisiae* and *C. elegans* obtained by SimLoRD [23], which is a read generation software mimicking the error pattern of PacBio sequencing.

All of our read sets had a genome coverage of 40x. *A. thaliana* reads were chosen from the larger set of reads from longest to shortest until we got to the target coverage. Information about the reference genomes and data sets can be seen in Table 1.

**Table 1:** Information about the data used in our experiments. Mitochondrial and chloroplast DNA are excluded

**Data sets**	*S. cerevisiae*	*C. elegans*	*A. thaliana*
Reference genome	NC_001133 -	NC_003279 -	LR215052 -
accession number	NC_001148	NC_003284	LR215056
Reference genome length [Mbp]	12.07	100.27	118.06
Number of chromosomes	16	6	5
Average chromosome length [Mbp]	0.75	16.71	23.61
Number of reads	58 806	488 554	213 113
Total length of reads [Mbp]	481.50	4 013.94	4 787.08
Average read length [Kbp]	8.19	8.22	22.46

For each of the genomes, we simulated a genome-wide optical map by finding the locations of the cut sites on the reference genome and then *in silico* digesting the genomes at these locations. We used the restriction enzyme XhoI in our simulations which recognizes the cut site ‘CTCGAG’.

### Error correction

To error correct each read set, CONSENT [22] was executed on a machine with 12 cores (2.2 GHz) and 64 GB memory. Table 2 shows the runtime and memory usage of CONSENT on each data set.

**Table 2:** CONSENT error correction results

**Error correction**	*S. cerevisiae*	*C. elegans*	*A. thaliana*
Time [hh:mm:ss]	04:13:58	21:16:15	76:33:52
Max. resident set size [MB]	6 535	9 027	22 714

### Experiments on read coloring

We can make many different decisions regarding how the reads will be colored, and the optimal settings are not obvious. For this reason, we experimented with different options to determine how reads should be colored to obtain the most promising results.

We defined the following four key steps in our read coloring process:

1. Align pre-coloring contig optical maps to reference optical map.
2. Align reads to the pre-coloring contigs.
3. Determine the regions each read covers in the pre-coloring contigs.
4. Remove possible gaps in the coloring.

Below we describe each of the steps in detail.

First, we align pre-coloring contig optical maps to reference genome optical map using the alignment tool by Valouev et al. [8], which will be referred to as VM from here on. VM outputs an alignment for each contig optical map with a quality score (s-score), and we need to determine how good the alignment should be for it to be trustworthy enough to be used in coloring. We experimented with two options here, either use all alignments (s-score threshold 0) or require a moderate amount of mapping quality (s-score threshold 15).

We also align reads to the pre-coloring contigs, which is done with minimap2 [24]. Minimap2 produces alignments, and among various statistics the number of matching bases between the read and the contig. We decided to try out three different ways to determine which alignments are suitable for coloring. (i) Use the alignment to a colored contig with the most matching bases, if one exists. (ii) Use the alignment to the contig with the most matching bases. If it is not colored, do not color the read. (iii) Use the alignment to a colored contig with the most matching bases, if the number of matching bases is at least *t*·*len*(*r*), where *t*=0.8 and *len*(*r*) is the read length.

The alignment can leave some of the bases at the ends of a read outside the aligning section. For the coloring, we could either decide to only consider the aligning part of the read or extend the alignment site in the contig with the non-aligning tails of the read.

Colors of the reads provide approximate information about their relative positions. Some regions of the genome can be poorly covered by the pre-coloring contigs. The regions might not appear in the contigs at all, or contain too many errors. Reads are not aligned to such regions, thus some of the possible colors do not appear in the reads at all. These color gaps can be fixed by dropping out the unused colors and readjusting the numbering of the used colors. We could either let the colors be as they are, or drop out the missing ones.

We ran experiments on the *C. elegans* data set to determine how each of these steps should be handled to get the most promising results. The results of the experiments can be seen in Table 3. In the end, we decided to adopt the following set of coloring options for our method.

**Table 3:** Comparison of *C. elegans* post-coloring contig assembly results with different read coloring schemes. Results are obtained by running Quality Assessment Tool for Genome Assemblies, QUAST 5.0.2, on the contigs and reference genome with ‘–large’ argument

**C. elegans pre-coloring contig results using miniasm**							
Minimum	Read	Extended	Adjusted	Number of	Length of	Mis-	NGA50
s-score	coloring	coloring	coloring	contigs	contigs [Kbp]	assemblies	[Kbp]
-	-	-	-	111	100 430	11	2 656
**C. elegans post-coloring contig results using**OpticalKermit**with color propagation**							
Minimum	Read	Extended	Adjusted	Number of	Length of	Mis-	NGA50
s-score	coloring	coloring	coloring	contigs	contigs [Kbp]	assemblies	[Kbp]
0	Only best	No	No	123	97 709	6	2 868
0	Best colored	No	No	162	97 408	6	1 741
0	Best hi-scoring	No	No	150	98 341	8	2 868
0	Only best	Yes	No	123	97 709	6	2 868
0	Best colored	Yes	No	162	97 408	6	1 741
0	Best hi-scoring	Yes	No	151	98 368	8	2 868
15	Only best	No	No	119	97 562	6	2 868
15	Best colored	No	No	157	97 274	6	1 741
15	Best hi-scoring	No	No	151	98 320	8	2 868
15	Only best	Yes	No	119	97 562	6	2 868
15	Best colored	Yes	No	157	97 274	6	1 741
15	Best hi-scoring	Yes	No	152	98 347	10	2 868
0	Only best	No	Yes	119	97 713	7	2 868
0	Best colored	No	Yes	162	97 434	6	1 741
0	Best hi-scoring	No	Yes	148	98 375	8	2 868
0	Only best	Yes	Yes	119	97 713	7	2 868
0	Best colored	Yes	Yes	162	97 434	6	1 741
0	Best hi-scoring	Yes	Yes	152	98 421	8	2 868
15	Only best	No	Yes	115	97 566	7	2 868
15	Best colored	No	Yes	157	97 300	6	1 741
15	Best hi-scoring	No	Yes	149	98 354	10	2 868
15	Only best	Yes	Yes	115	97 566	7	2 868
15	Best colored	Yes	Yes	157	97 300	6	1 741
15	Best hi-scoring	Yes	Yes	153	98 399	10	2 868
**C. elegans post-coloring contig results using**OpticalKermit**without color propagation**							
Minimum	Read	Extended	Adjusted	Number of	Length of	Mis-	NGA50
s-score	coloring	coloring	coloring	contigs	contigs [Kbp]	assemblies	[Kbp]
0	Only best	No	No	93	100 192	8	2 868
0	Best colored	No	No	92	100 175	11	2 683
0	Best hi-scoring	No	No	131	100 022	10	2 868
0	Only best	Yes	No	93	100 192	8	2 868
0	Best colored	Yes	No	92	100 175	11	2 683
0	Best hi-scoring	Yes	No	130	100 019	10	2 868
15	Only best	No	No	102	100 414	10	2 868
15	Best colored	No	No	96	100 270	12	2 683
15	Best hi-scoring	No	No	131	99 982	12	2 868
15	Only best	Yes	No	102	100 414	10	2 868
15	Best colored	Yes	No	96	100 270	12	2 683
15	Best hi-scoring	Yes	No	130	99 979	12	2 868
0	Only best	No	Yes	94	100 215	8	**3 028**
0	Best colored	No	Yes	92	100 200	11	2 683
0	Best hi-scoring	No	Yes	133	100 077	10	2 868
0	Only best	Yes	Yes	94	100 215	8	**3 028**
0	Best colored	Yes	Yes	92	100 200	11	2 683
0	Best hi-scoring	Yes	Yes	133	100 086	10	2 868
15	Only best	No	Yes	103	100 436	10	**3 028**
15	Best colored	No	Yes	96	100 296	12	2 683
15	Best hi-scoring	No	Yes	133	100 037	12	2 868
15	Only best	Yes	Yes	103	100 436	10	**3 028**
15	Best colored	Yes	Yes	96	100 296	12	2 683
15	Best hi-scoring	Yes	Yes	133	100 046	12	2 868

- No minimum quality score (s-score) in contig coloring.
- A read is colored based on the best aligning contig only.
- Read coloring regions are extended beyond the aligning regions.
- Read colors are adjusted so that the used colors do not contain gaps.

### Assembly results

We assembled the three data sets, *S. cerevisiae*, *C. elegans* and *A. thaliana*. The assemblies were run on a machine with IntelⓇCore^TM^ i5-8250U CPU @ 1.60GHz ×8 processor and 15.5 GB of memory. All three assemblies were evaluated using QUAST (Quality Assessment Tool for Genome Assemblies) [25]. Table 4 shows the results for pre- and post-coloring assemblies. Also miniasm has been run on error-corrected reads. These experiments have been run with a newer version of miniasm than the coloring experiment of the previous section and thus there are small discrepancies between the results in Tables 3 and 4.

**Table 4:** Pre- and post-coloring contig assembly results using miniasm, OPTICALKERMIT and Canu. Canu (CONSENT) shows the assembly results of Canu when executed on the CONSENT corrected reads and Canu shows the assembly results when Canu is executed on the original reads which are first corrected by Canu

**S. cerevisiae contig assembly results**						
	Number	Number	Length of	Length of		
	of	of >50Kbp	contigs	>50Kbp contigs	Mis-	NGA50
	contigs	contigs	[Kbp]	[Kbp]	assemblies	[Kbp]
miniasm	26	21	12 050	11 987	4	777
OpticalKermit	23	18	12 031	11 968	4	810
Canu (CONSENT)	24	16	12 111	11 970	3	810
Canu	22	16	12 039	11 984	1	922
**C. elegans contig assembly results**						
	Number	Number	Length of	Length of		
	of	of >50Kbp	contigs	>50Kbp contigs	Mis-	NGA50
	contigs	contigs	[Kbp]	[Kbp]	assemblies	[Kbp]
miniasm	110	74	100 398	99 737	8	2 641
OpticalKermit	87	57	100 241	99 706	7	3 568
Canu (CONSENT)	276	191	100 080	99 035	7	807
Canu	20	12	100 261	100 080	1	10 769
**A. thaliana contig assembly results**						
	Number	Number	Length of	Length of		
	of	of >50Kbp	contigs	>50Kbp contigs	Mis-	NGA50
	contigs	contigs	[Kbp]	[Kbp]	assemblies	[Kbp]
miniasm	532	121	133 522	120 191	61	1 469
OpticalKermit	230	87	123 447	119 034	59	1 587
Canu (CONSENT)	3 482	390	79 897	30 616	85	20
Canu	459	314	118 772	114 913	100	592

Additionally, we compared OPTICALKERMIT against Canu assembler [7]. We ran Canu on the CONSENT corrected reads with the correction of the reads by Canu disabled. We also ran Canu on the original reads allowing it to correct the reads by its own method. The assembly statistics of Canu are also shown in Table 4. Table 5 shows the runtime and memory usage statistics of miniasm, OPTICALKERMIT, and Canu.

**Table 5:** Memory and time usage of the different tools. Miniasm, OPTICALKERMIT, and Canu (CONSENT) use CONSENT corrected reads and the time and memory used for the correction is not included in the statistics. The row titled Canu shows the memory and time usage of Canu with the original reads. Although in this case we allowed Canu to use its own read correction, here we show the memory usage and runtime of Canu without the correction step for fairer comparison. The correction step of Canu took 2 h, 22 h, and 29 h of time and 5 GB, 11 GB, and 12 GB memory on *S. cerevisiae*, *C. elegans*, and *A. thaliana*, respectively

***S. cerevisiae**** assembly memory and time usage*		
	Memory [MB]	Time [hh:mm:ss]
miniasm	4 542	00:06:05
OpticalKermit	4 542	00:06:43
Canu (CONSENT)	2 268	00:34:31
Canu	2 247	00:33:14
***C. elegans**** assembly memory and time usage*		
	Memory [MB]	Time [hh:mm:ss]
miniasm	14 592	00:55:18
OpticalKermit	14 592	01:08:18
Canu (CONSENT)	7 970	06:55:09
Canu	11 713	07:28:55
***A. thaliana****assembly memory and time usage*		
	Memory [MB]	Time [hh:mm:ss]
miniasm	15 285	06:09:29
OpticalKermit	15 413	07:06:39
Canu (CONSENT)	8 255	17:43:56
Canu	10 901	23:53:23

During contig assembly, OPTICALKERMIT does not consider reads that have not been colored. Thus it is possible that merely leaving out these reads improves the assembly. To investigate this, we performed the following experiment on the *C. elegans* data set. Instead of coloring the reads according to the genome-wide optical map, we colored each read that aligned to a contig with the same color unique to the contig, and ran OPTICALKERMIT on this unicolored read set. Table 6 shows the results of this experiment. We see that the results for using unicolored reads are practically the same as the pre-colored assembly, i.e. running miniasm directly on the non-colored reads. Thus the improvements that we see in Table 4 are due to using the genome-wide optical map to guide the assembly.

**Table 6:** *C. elegans* assembly results with normally colored and unicolored contigs, with miniasm pre-coloring assembly results for comparison. The most promising coloring parameters were used without OPTICALKERMIT’s color propagation. The results for unicolored OPTICALKERMIT are practically the same as the results of the pre-coloring assembly

**C. elegans contig assembly results with**OpticalKermit**(no color propagation)**								
			Number	Number	Length of	Length of		
Assembler	Coloring	Propagation	of	of >50Kbp	contigs	>50Kbp contigs	Mis-	NGA50
			contigs	contigs	[Kbp]	[Kbp]	assemblies	[Kbp]
OpticalKermit	Normal	No	94	61	100 215	99 637	8	3 028
OpticalKermit	Normal	Yes	119	55	97 713	96 108	7	2 868
OpticalKermit	Unicolored	No	111	75	100 430	99 770	11	2 656
OpticalKermit	Unicolored	Yes	109	74	100 394	99 758	11	2 656
miniasm	-	-	111	75	100 430	99 770	11	2 656

Our assembly pipeline consists of the following steps: pre-coloring contig assembly, optical map generation and alignment, read-to-contig aligning, read coloring, and post-coloring contig assembly. Table 7 has the time and memory consumptions of each of these steps.

**Table 7:** Memory and time usage of different phases of OPTICALKERMIT

**S. cerevisiae assembly memory and time usage**			
	Memory [MB]	Time [hh:mm:ss]	Time [%]
Read-to-read alignment (minimap2)	4 542	00:05:58	88.8
Pre-coloring assembly (miniasm+awk)	179	00:00:07	1.7
Optical map alignment (create + alignment)	44	00:00:05	1.2
Read-to-contig alignment (minimap2)	561	00:00:23	5.7
Read coloring (contig+read coloring)	112	00:00:03	0.7
Post-coloring assembly (Kermit+awk)	179	00:00:07	1.7
Maximum memory / Total time	4 542	00:06:43	100.0
**C. elegans assembly memory and time usage**			
	Memory [MB]	Time [hh:mm:ss]	Time [%]
Read-to-read alignment (minimap2)	14 592	00:53:19	78.1
Pre-coloring assembly (miniasm+awk)	1 131	00:01:59	2.9
Optical map alignment (create + alignment)	818	00:05:08	7.5
Read-to-contig alignment (minimap2)	2 057	00:03:30	5.1
Read coloring (contig+read coloring)	1 613	00:02:30	3.7
Post-coloring assembly (Kermit+awk)	1 131	00:01:52	2.7
Maximum memory / Total time	14 592	01:08:18	100.0
**A. thaliana assembly memory and time usage**			
	Memory [MB]	Time [hh:mm:ss]	Time [%]
Read-to-read alignment (minimap2)	15 210	05:52:37	82.6
Pre-coloring assembly (miniasm+awk)	15 285	00:16:52	4.0
Optical map alignment (create + alignment)	1 597	00:12:16	2.9
Read-to-contigs alignment (minimap2)	2 479	00:19:57	4.7
Read coloring (contig+read coloring)	3 952	00:01:39	0.4
Post-coloring assembly (Kermit+awk)	15 413	00:23:18	5.5
Maximum memory / Total time	15 413	07:06:39	100.0

## Discussion

In our experiments with simulated data, OPTICALKERMIT produced longer contigs than an unguided assembly, i.e. the miniasm [26] pre-coloring assembly. NGA50 statistic improved and the number of contigs decreased with both simulated and real reads. This suggests that OPTICALKERMIT is able to connect some contigs that would otherwise be left separate. From the technical point of view, this means that OPTICALKERMIT is able to extend non-branching paths in the assembly graph by deleting spurious edges. The total length of the post-coloring contigs did not drop a considerable amount from the pre-coloring contigs, so the original genome is still extensively covered. On the *S. cerevisiae* genome, the total length of the contigs got slightly further away from the reference length. On the other hand, on the *C. elegans* genome we actually got closer to the reference genome length. The number of misassemblies stayed the same or even dropped on the simulated data sets, which is a positive sign.

With the real *A. thaliana* reads, the total number of contigs produced by OPTICALKERMIT decreased drastically and their total length dropped closer to the total length of the reference as compared to the unguided assembly by miniasm. The NGA50 statistic also increased as compared to the pre-coloring assembly and the number of misassemblies decreased. Therefore, these experiments show that OPTICALKERMIT is a promising method for improving genome assemblies also on real read data.

The comparison against Canu gave mixed results. In general, Canu performed better with its own error correction method than on CONSENT corrected reads. On simulated data, the assemblies produced by Canu using its own error correction method were both more continuous as evidenced by a higher NGA50 value and had fewer misassemblies than contigs produced by OPTICALKERMIT. However, on the real *A. thaliana* data set, the NGA50 of contigs produced by OPTICALKERMIT is almost thrice that of contigs produced by Canu with its own error correction method and also the number of misassemblies is lower for OPTICALKERMIT. Simulated data is usually easier for assemblers due to the inability of read simulators to incorporate all artifacts produced by real sequencing machines. Thus we find the good performance of OPTICALKERMIT on the real *A. thaliana* reads encouraging.

Because real genome-wide optical maps are not readily available for model species with an accurate and complete reference genome, all our experiments used a simulated genome-wide optical maps. As future work, it would thus be interesting to see how OPTICALKERMIT performs with real optical mapping data.

Table 7 shows that the time and memory consumptions of OPTICALKERMIT do not increase remarkably compared to the unguided approach. The most memory-intensive tasks are the read-to-read mapping and the assemblies themselves, which are required in both guided and unguided approaches. The most time-consuming step is the read-to-read alignment with minimap2 [24]. Even in the worst case, the addition of read coloring and OPTICALKERMIT assembly was responsible for less than 20% of the total used time. As compared to Canu, OPTICALKERMIT uses more memory but is much faster. However, the correction step of Canu is faster than CONSENT which has been used to correct the reads for OPTICALKERMIT.

Even though the performance regarding time and memory did not suffer too much, we still need to remember that before everything else all the reads were corrected with CONSENT [22]. Looking at Table 2 it becomes very obvious that this is actually the step that was responsible for the majority of the running time, and also requires a large amount of memory. However, to produce a polished final assembly either the reads need to be corrected or the final contigs polished both for OPTICALKERMIT and miniasm.

Using optical maps during assembly is a research area that has not been focused on intensively, but has gathered interest in recent years. A few example programs that utilize optical maps during genome assembly can already be found, like the aforementioned AGORA [4] and KOOTA [5]. KOOTA is not very competitive as an assembler compared to the other state-of-the-art assemblers, having a weaker N50 score according to the authors. This was due to the emphasis of the research being on using optical maps to simplify de Bruijn graphs in the genome assembly, not developing a sophisticated traversal algorithm for it. AGORA assembler also utilized optical maps with de Bruijn graphs, and it performed quite well as an assembler, but it was only tested with error-free reads of short bacterial genomes. Here we have developed a guided assembly pipeline that utilizes optical maps during contig assembly, generates high-quality contigs that are competitive with other assemblers, and is also applicable for genomes more complex than just bacterial ones.

## Conclusions

We have presented a new genome assembly pipeline to utilize optical maps automatically during the assembly process. First, the long reads are corrected with CONSENT [22]. The corrected reads are then given to miniasm [26] assembler to produce initial pre-coloring contigs. Next, optical maps of the contigs are generated computationally and aligned with the optical maps of the reference genome chromosomes using VM [8]. The contigs are also aligned with the corrected reads with minimap2 [24]. The optical map alignment data is then used with the read-to-contig alignments to approximate the origin locations of the reads within the genome. Location approximations are represented by coloring the reads. Finally, these colored reads are given to Kermit [6] assembler to produce the post-coloring contigs as our final assembly product.

The implementation of OPTICALKERMIT is modular. Most of the tasks in the pipeline could be performed by any tool that can produce the output in the required standard format such as FASTA for reads and contigs or PAF for alignments. More specifically, read error correction, pre-coloring assembly, aligning contigs to the optical map, and aligning reads to contigs could be performed by any appropriate tool. The post-coloring assembly phase is more tightly integrated with minimap2 and would thus require further engineering to adapt for a different assembler.

Our method is complementary to scaffolding tools using optical mapping data such as OMGS [15] and SEWINGMACHINE [27]. OPTICALKERMIT can improve the length of contigs, whereas scaffolders order and orient the contigs without modifying the contigs themselves. Thus applying both OPTICALKERMIT and a scaffolder using optical mapping data could improve both contig and scaffold statistics of an assembly. Alternatively, as further work, OPTICALKERMIT could be extended to a scaffolder by leveraging the colors assigned to contigs and estimating gaps between contigs based on the optical maps.

Our OPTICALKERMIT assembly pipeline is able to handle more complex genomes even with real reads and performs competitively to its unguided counterpart assembler miniasm. In the case of real reads, OPTICALKERMIT is also able to produce more contiguous contigs compared to another state-of-the-art assembler Canu. In summary, the usage of optical maps seems promising during contig assembly, and more research into this subject is warranted. We have shown that our proposed assembly scheme can be a viable option, and could still be improved with further development.

## Methods

### Error correction

We use third generation Pacific Biosciences long reads which have a high error rate. Without error correction or contig polishing, the error rate would stay the same for the final assembly. We decided to address this problem at the beginning of the pipeline by correcting the reads. Error correction not only improves de novo assembly results, but in our approach it also helps the guided assembly by improving the quality of the in silico optical maps. Insertions, deletions, and substitutions affect the observed cut sites, and correcting these errors helps lost cut sites to be restored and false cut sites to disappear.

We use CONSENT [22] for correcting the sequencing errors. CONSENT is a fairly recent error correction tool shown to perform well as compared to the other state-of-the-art self-correction methods. One weakness of CONSENT is that its running time is noticeably longer in comparison. We decided this is acceptable because the throughput and resulting error rate are good.

### Pre-coloring contig assembly

The pre-coloring assembler of our choice is miniasm [26], which Kermit assembler [6] is also based on. Before the miniasm assembly can be run, we need to use a program that aligns reads to each other. A natural choice for this is minimap2 [24], a successor to the minimap mapping program introduced in the miniasm article.

Minimap2 finds overlaps between the input reads, and based on the strongest overlaps, miniasm builds an overlap graph. Miniasm skips the consensus step in the OLC (overlap, layout, consensus) assembly paradigm, and only looks for non-branching paths, unitigs, in the graph. Here we call the initial miniasm assembly results pre-coloring contigs.

### Optical map alignment

We express the relative location information between reads, which Kermit [6] requires, by coloring them with the help of optical maps. The optical maps of the in silico digested reads often have very few fragments, therefore they are not suitable for reliable mapping. On the other hand, optical maps of the pre-coloring contigs are easier to align since they are considerably longer leading to more convincing alignments. This is the reason we produce pre-coloring contigs and use them to color the reads.

First, the in silico digested pre-coloring contigs from the unguided miniasm assembly are aligned to the reference genome optical map. After the fragment-to-fragment alignments between reference and pre-coloring contig optical maps are known, the contig optical map fragments can be colored. Next, the reads are aligned with the colored pre-coloring contigs. The alignments and the contig color information can be used to color the reads.

We perform optical map alignment with the alignment tool VM [8], which takes errors occurring in restriction digestion into consideration. As suitable real optical maps were hard to find, we had to use in silico optical maps. Thus our data does not actually contain restriction digestion optical maps, which VM is specialized in. We assumed it would still perform well with computationally generated optical maps, which our experimental results confirmed.

VM calculates two mapping quality scores, the likelihood score used in their model called s-score, and a heuristic score proposed in [28] called t-score. The s-score is shown to work noticeably better with real-world optical maps, while with simulated optical the improvements were minor. As there was no downside in using the s-score, we decided to only consider it when determining how contigs should be colored. Our experimental results suggested that in the best combination of coloring options we should consider every mapping even if the s-score was low.

### Contig coloring

The output of VM [8] tells us which cuts of the query optical map align to which cuts of the reference optical map. Another way to interpret this is to consider it as fragments mapping to each other. The blocks of fragments between contig and reference optical maps also align to each other similarly to the cut sites. There are two obvious ways to color the contigs, either color the cuts or color the fragments. We decided to go with the fragment coloring route, but using cut sites seems like a valid approach too.

We number the fragments of the reference optical maps from 0 to *n*−1 where *n* is the number of fragments. To separate the fragments of different chromosomes in the reference optical map, we add *k*·*s* to the number of the fragment, where *k* is the number of the chromosome and *s* is an appropriate power of ten so that optical maps of two different chromosomes do not share a color. This guarantees that every color occurs only once in the whole reference optical map. The resulting fragment numbers of the reference optical map represent the colors in our coloring scheme. Fragments of the pre-coloring contig optical maps are colored based on their mapping with reference optical map fragments. There are four different fragment mapping cases listed below:

1. one to one
2. one to many
3. many to one
4. many to many

**Fig. 3:**
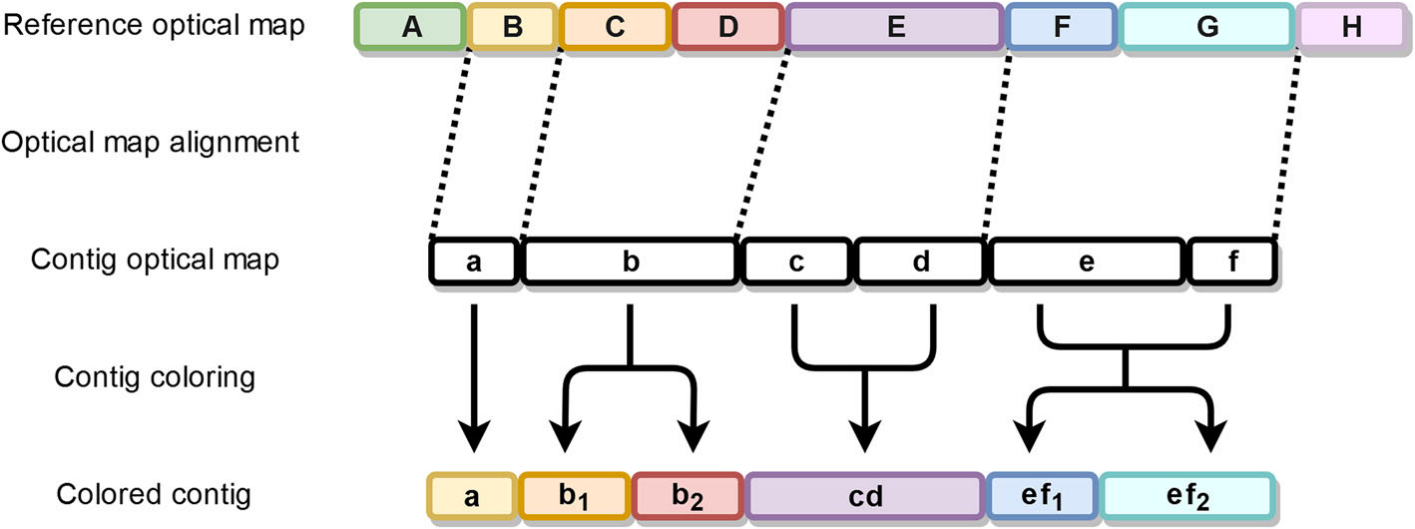
Contig coloring example illustrating four different fragment alignment cases. **Case 1:** Contig fragment *a* aligns to reference fragment *B*, and is colored with its color. **Case 2:** Contig fragment *b* aligns to reference fragments *C* and *D*. It is split into two fragments *b*_1_ and *b*_2_ whose total length is equal to the length of *b*, keeping the proportions of *C* and *D*. Fragment *b*_1_ is colored with the color of fragment *C*, and *b*_2_ with the color of *D*. **Case 3:** Contig fragments *c* and *d* align to reference fragment *E*. Fragments *c* and *d* are merged together into one fragment *cd* which is colored with the color of fragment *E*. **Case 4:** Contig fragments *e* and *f* align to reference fragments *F* and *G*. Fragments *e* and *f* are transformed into two fragments *ef*_1_ and *ef*_2_ with the total length of *e* and *f*, keeping the proportions of fragments *F* and *G*. Fragment *ef*_1_ is colored with the color of fragment *F*, and *ef*_2_ with the color of *G*

In the first case, the query fragment (contig optical map fragment) is simply colored with the matching target fragment (reference optical map fragment) color. In the second case, the query fragment is split into as many fragments as there are target fragments. The new fragment lengths are relative to the target fragment sizes, but their total length is that of the query fragment. These new fragments replace the old fragment, and they are colored with the colors of their respective target fragments. In the third case, all query fragments are colored with the color of the single target fragment. The fourth case is similar to the second case, but now the total length of the new fragments is the total length of all query fragments. Figure 3 illustrates these four different coloring cases.

These coloring cases can be generalized into a single coloring rule. Suppose *N* contig fragments with total length *n* map to *M* reference fragments with total length *m*. Create new fragments by taking the *M* reference fragments and dividing their lengths by *m* and multiplying by *n*. Keep the colors the same as in the original *M* reference fragments and replace the original *N* contig fragments with these new colored fragments.

VM gives at most one alignment for each query optical map. Even though VM provides scores for each alignment, our experiments suggest that it would be beneficial to ignore the scores and color every pre-coloring contig that can be aligned to the reference optical map.

### Read coloring

Now we are ready to color the corrected reads. We start by aligning the reads to the pre-coloring contigs. This is done with minimap2 [24] to keep our aligning processes consistent, since this program is also used in pre-coloring contig assembly with miniasm [26], read correcting with CONSENT [22], and post-coloring contig assembly with Kermit [6].

Minimap2 can produce multiple possible alignments for a single read. We decided each read should be colored based on one alignment only, and the intuitive solution is to use the ‘best’ alignment for coloring. After the initial experiments seen in Table 3, we decided on a simple heuristic. A read is colored based on the alignment with the greatest number of matching bases between it and the aligning section of the contig. If the number of matching bases was less than 80% of the whole length of the read, the read was left uncolored.

For each alignment, the start and end positions in a contig are known, and they are used to determine which colors of the contig are used to color the read. For example, suppose the aligning section in the contig starts from position *i* and ends at position *j*. We begin to add the lengths of the contig fragments together one by one. The first fragment *n*, for which the sum of the lengths of the contig fragments [0,*n*] is greater than *i* is chosen as the starting fragment i.e. the starting color. Similarly, the first fragment *m*, for which the sum of the lengths of the contig fragments [0,*m*] is greater than *j*, is used as the end fragment i.e. the end color. As Kermit only uses the start and end colors of a read, this coloring scheme suffices.

**Fig. 4:**
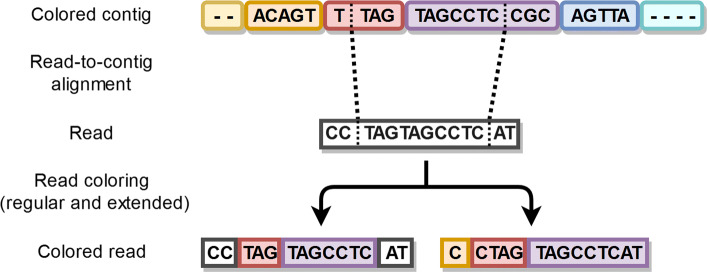
Simplified example of the two different cases of read coloring when a reasonably strong alignment with a colored contig is found. **Case 1:** Left hand side coloring is based on the aligning segment only. **Case 2:** Right hand side coloring is based on the aligning segment extended with the lengths of the non-aligning sections of the read

We deliberated if the beginning and end parts of the reads that are left outside of the aligning section should be used to extend the coloring beyond the aligning section in the contig. For example, suppose the alignment in the contig starts again at position *i* and ends at position *j*. Additionally, suppose we also know that there is a sequence of length *a* in the beginning of the aligning section of the read, and a sequence of length *b* at the end of aligning section of the read. The question is, would it be beneficial to adjust the starting position of the aligning section in the contig to start at *i*−*a* and to end at *j*+*b* to possibly extend read colors. Figure 4 shows how extending could affect the coloring. As we expected, the coloring experiments revealed that this does not greatly affect the quality of the resulting post-coloring contigs. If the extensions would have a huge impact on the coloring, it would suggest that the aligning section itself would be short, which should not be possible because we are requiring it to have at least 80% matching bases. Nevertheless, we decided to include the extensions in our pipeline.

After the reads are colored, some of the available colors in the contigs can be completely unused by the reads. In other words, none of the read alignments overlapped with the fragments corresponding to the missing colors. To be clear, colors between the start and end colors of a read are also considered as used. It is possible that two reads are in reality physically close to each other, even though there is a color gap between them due to inaccurate alignments for example. This might affect the performance of Kermit negatively, so we decided to shift the colors so that there were no missing colors.

As a simplified example, suppose all the reads used colors {1,2,4,7,8,10}, while the colors appearing in the reference were {1,2,3,4,5,6,7,8,9,10}. These read colors would then be mapped to consecutive colors {1,2,3,4,5,6} in their respective order; 1→1, 2→2, 4→3, 7→4, 8→5, 10→6. Then the already colored reads would be recolored according to these mappings. They would still keep their respective order, but the gaps caused by unused colors get removed. Colors were only shifted within a single chromosome’s colors i.e. the *s*·*k* term in the colors was left untouched. We ran experiments with and without this adjustment to determine its effects, and the results suggest that readjusting the colors is beneficial.

### Post-coloring contig assembly

After all the data is gathered and processed to get the corrected and colored reads, we can start assembling post-coloring contigs. Our OPTICALKERMIT assembly pipeline uses the Kermit assembler [6] to do this. Kermit works very similarly to the miniasm assembler [26], the difference being that during the layout step Kermit cleans the assembly graph based on the given colorings.

Kermit starts by building an overlap graph with the help of minimap2 [24] alignments. The same read-to-read alignments that were used during the pre-coloring assembly can be used here. Each vertex of the graph represents a DNA sequence, and we know which read is responsible for it. Since the reads have been colored, the vertices can also be colored accordingly, leading to a colored overlap graph.

As with miniasm, a unitig is again defined as a maximal non-branching path in the overlap graph. However, with the vertex color information, we can alter the paths that are used to build the unitig. An edge from vertex *v*_*i*_ to vertex *v*_*i*+1_ means the corresponding sequences overlap, and can be merged together to be used as a part of a unitig. Some of the connections are bound to be erroneous, which can be detected with the help of the colors.

Suppose an edge (*v*_*i*_,*v*_*i*+1_) exists in the graph, but the color number of vertex *v*_*i*_ was larger than that of vertex *v*_*i*+1_. The color information suggests that *v*_*i*_ should appear after *v*_*i*+1_, but the edge says they are connected together in the reverse order. We trust the coloring information acquired from the optical maps more than the overlap edge, and it is removed completely from the graph. Even if the order of the colors is correct, their distance can cause suspicion. If the colors are very far apart, we can deduce the vertices should not be connected with an edge, and such edges are also removed from the graph. These kind of contradicting edges are called *inconsistent* edges.

A *consistent* edge (*v*_*i*_,*v*_*i*+1_) is an edge such that at least one of the colors of vertex *v*_*i*+1_ is equal to or exactly one greater than at least one of the colors in vertex *v*_*i*_. By default Kermit only allows the colors to differ by at most one which is the option we used, but this restriction can be adjusted by the user. All edges that do not follow this requirement are discarded. This way we cannot take a path with a huge gap or where some of the sequences are in the wrong order.

Some of the reads might be left uncolored due to difficulties and uncertainties during the coloring step. An edge that is connected to an uncolored vertex would be automatically removed, but Kermit alleviates this problem of uncolored vertices by allowing the colors to propagate. An uncolored vertex is assigned all colors of the vertices that are reachable from it by traveling only through uncolored vertices. There is a limit on how far the colors should propagate, since allowing the colors to flow as far as possible would most likely lead to incorrect colorings. By default Kermit allows the colors to propagate through five vertices, but this can again be changed to the user’s liking. If propagated colors have some missing colors between them, the vertex is deleted completely from the graph because the propagated coloring is not coherent which makes us suspicious of its correctness. We experimented with the default option and the no propagation option, and the results can be seen in Table 3. The best read coloring option combination was found when Kermit’s color propagation was disabled, which is the reason we did not allow the colors to propagate in the final assembly pipeline.

After inconsistent edges are removed, Kermit starts looking for the unitigs. Instead of finding all maximal non-branching paths, we automatically find all maximal non-branching *rainbow paths*. A rainbow path is like a normal path, but all the colors must appear in increasing order and only once. Kermit loosens this condition by allowing consecutive vertices to use the same color on a path. The removal of all inconsistent edges guarantees that every path we find is also a rainbow path. Kermit outputs these maximal non-branching rainbow path unitigs as our final assembly product.

## Data Availability

OpticalKermit is freely available at https://github.com/Denopia/kermit-optical-maps. *S. cerevisiae* reference genome is available at https://www.ncbi.nlm.nih.gov/genome/15?genome_assembly_id=22535. *C. elegans* reference genome is available at https://www.ncbi.nlm.nih.gov/genome/41?genome_assembly_id=43998. *A. thaliana* reference genome is available at https://www.ncbi.nlm.nih.gov/genome/4?genome_assembly_id=454618. *A. thaliana* reads are available at https://downloads.pacbcloud.com/public/SequelData/ArabidopsisDemoData.

## Abbreviations

GB: Gigabytes
Kbp: Kilobasepairs
MB: Megabytes
OLC: Overlap-layout-consensus
SNV: Single nucleotide variation
VM: Valouev et al. mapper
